# Effects of rumen-protected glucose supplementation strategies on growth performance, colonic fermentation, and meat quality in Simmental beef cattle

**DOI:** 10.5713/ab.260255

**Published:** 2026-06-01

**Authors:** Zhenyu Liu, Sikai Wang, Bing Bai, Haihao Huang, Xianchao Guan, Lingyan Li

**Affiliations:** 1 College of Animal Science and Veterinary Medicine, Heilongjiang Bayi Agricultural University, Key Laboratory of Low-carbon Green Agriculture in Northeastern China, Ministry of Agriculture and Rural Affairs, Daqing, China

**Keywords:** Colonic Fermentation, Growth Performance, Intramuscular Fat, Rumen-protected Glucose, Simmental Beef Cattle

## Abstract

**Objective:**

This study was conducted to evaluate the effects of supplementation strategies containing rumen-protected glucose (RPG) on growth performance, colonic fermentation characteristics, bacterial community structure, carcass traits, and meat quality in Simmental beef cattle.

**Methods:**

Thirty Simmental bulls with an initial body weighing 578.4±14.7 kg were randomly assigned to three dietary treatments for 90 d, with 10 animals per treatment. All animals received the same basal total mixed ration, to which the treatment supplements were added. The treatments were CON, unprotected glucose (150 g/d) plus coating fat (150 g/d); T1, RPG (100 g/d) plus unprotected glucose (100 g/d) and coating fat (100 g/d); and T2, RPG (300 g/d). Growth performance was recorded throughout the trial. At the end of the experiment, colonic digesta were collected for fermentation and bacterial community analyses, and carcass traits and meat quality were evaluated after slaughter.

**Results:**

T1 and T2 exhibited higher average daily gains (ADGs) compared to CON (p<0.05), while feed efficiency differed marginally among treatments (p = 0.049). In colonic digesta, propionate concentrations were higher in T1 and T2 than in CON, and butyrate concentration was higher in T2 than in CON and T1 (p<0.05). Alpha diversity was not affected by treatment; however, unweighted UniFrac-based principal coordinate analysis showed differences in the overall colonic bacterial community structure among treatments. At the phylum and genus levels, several taxa showed nominal differences based on raw p values, but these were not retained after false discovery rate correction. Hot carcass weight and meat weight were greater in T1 and T2 than in CON (p<0.05). Intramuscular fat content was higher in T2 than in CON (p<0.05), whereas most other meat quality traits were not significantly affected.

**Conclusion:**

Under the conditions of this study, the supplementation strategies containing RPG were associated with increased overall ADG, hot carcass weight, and meat weight in Simmental beef cattle. These strategies were also associated with higher colonic propionate and butyrate levels, indicating changes in hindgut fermentation. The higher-RPG strategy (T2) was associated with increased intramuscular fat content.

## INTRODUCTION

Improving feed efficiency and carcass value while maintaining desirable meat quality is a central goal in finishing beef production. Nutritional approaches that enhance dietary energy utilization and regulate nutrient partitioning are therefore of considerable interest. Glucose plays a pivotal role in energy metabolism, and increasing absorbable glucose supply has been proposed as a strategy to support growth and potentially influence fat deposition and meat quality traits [1,2]. Furthermore, because growth-related traits in beef cattle are closely associated with later productive performance, nutritional strategies that improve weight gain efficiency and carcass development are of particular importance [3].

In ruminants, dietary carbohydrates are extensively fermented in the rumen, and volatile fatty acids (VFAs) provide the major share of metabolizable energy to the host [4]. Consequently, systemic glucose availability depends largely on gluconeogenesis from fermentation end-products rather than direct intestinal absorption of dietary glucose. Because of these physiological constraints, approaches that increase post-ruminal glucose supply either by increasing small intestinal starch digestion or by providing glucose in a rumen-protected form have received increasing attention [5,6]. Rumen-protected glucose (RPG) products are designed to bypass ruminal fermentation and deliver glucose to the small intestine, thereby increasing the proportion of glucose absorbed post-ruminally. However, responses to RPG may vary depending on dose and basal diet [7].

Shifting the site of carbohydrate utilization may also influence the hindgut ecosystem. Changes in the quantity and type of substrates reaching the large intestine can alter colonic fermentation and microbial community structure, potentially affecting VFA profiles. Although hindgut fermentation contributes less to total energy supply than ruminal fermentation, colonic VFAs and microbiota provide useful indicators of how dietary interventions redistribute fermentable substrates along the gastrointestinal tract and may provide mechanistic insight into observed production responses [8,9]. Recent reviews have highlighted that the ruminal and hindgut microbiota in beef cattle are associated not only with feed efficiency and methane production, but also with carcass characteristics and meat quality. However, nutritional strategies that can effectively integrate these responses remain limited [10]. Dietary interventions using alternative forage resources have been shown to influence ruminant performance, antioxidant capacity, immune function, and ruminal health, while host-related factors such as age and gender are also associated with variation in rumen fermentation characteristics and microbial community structure in cattle [11,12]. These findings further indicate that nutritional responses in ruminants should be evaluated in relation to both productive performance and gastrointestinal fermentation or microbial changes.

Therefore, this study investigated the effects of graded RPG supplementation on growth performance, colonic fermentation characteristics, colonic bacterial community structure, and carcass traits and meat quality in finishing Simmental bulls. To improve comparability among treatments, unprotected glucose and coating fat were included as compensatory supplements in the control and intermediate treatment groups. We hypothesized that supplementation strategies containing different proportions of RPG would be associated with differences in growth performance, hindgut fermentation, and microbial profiles, with potential implications for carcass characteristics and meat quality.

## MATERIALS AND METHODS

### Animals, experimental design, and management

Thirty Simmental bulls (initial body weight [BW], 578.4±14.7 kg) were used in a 90-d feeding trial. Bulls were blocked by initial BW and randomly assigned to one of three dietary treatments (n = 10 per treatment). Animals were maintained in a tie-stall housing system under standard management conditions with free access to water. Following a 7-d adaptation period, the experimental treatments were applied for 90 d.

Dietary treatments and supplementation levels are presented in Table 1, and the ingredient composition and formulated nutrient levels of the basal total mixed ration (TMR) are provided in Table 2. Bulls received one of the following treatments: CON, unprotected glucose (150 g/d) plus coating fat (150 g/d); T1, RPG (100 g/d) plus unprotected glucose (100 g/d) and coating fat (100 g/d); and T2, RPG (300 g/d) without additional unprotected glucose or coating fat. The RPG product consisted of glucose encapsulated with a fat-based coating at a 1:1 (wt/wt) ratio.

To improve comparability among treatments, unprotected glucose and coating fat were included in CON and T1 as compensatory supplements. Because the RPG product consisted of glucose and a fat-based coating at a 1:1 (wt/wt) ratio, this design helped reduce treatment differences associated with the coating component and facilitated comparison among supplementation strategies. Specifically, coating fat was added to account for the fat-based coating inherent to the RPG product, whereas unprotected glucose was included to offset part of the glucose supplied by RPG. All supplements were weighed daily and top-dressed onto the TMR to ensure complete consumption.

Bulls were fed the basal TMR ad libitum, with feed offered twice daily. Water was freely available throughout the experiment. Feed intake was recorded every 15 d to determine dry matter intake (DMI) during the experimental period.

### Growth performance measurements

Individual BW was recorded on day 0, 45, and 90 before the morning feeding. Average daily gain (ADG) was calculated as BW change divided by days on feed for each period (0–45, 46–90, and 0–90 d). Feed efficiency was expressed as ADG/DMI.

### Slaughter procedure and colonic digesta sampling

At the end of the feeding trial, bulls were slaughtered in accordance with routine commercial practices. Immediately after evisceration, colonic digesta were aseptically collected from the proximal, mid, and distal colon, pooled in equal amounts, thoroughly homogenized to generate one representative composite sample per animal and then portioned into aliquots. Aliquots designated for fermentation analyses were kept on ice during processing and stored at −80°C within approximately 1 h after sampling. Aliquots for microbial analysis were snap-frozen in liquid nitrogen and stored at −80°C until DNA extraction.

### Colonic fermentation parameters

Colonic digesta pH was measured immediately after sampling using a calibrated portable pH meter (Testo 206; Testo) by inserting the electrode directly into fresh digesta. For VFA determination, colonic digesta (1 g, wet weight) were diluted with 9 mL of distilled water (1:10, wt/vol), homogenized, and centrifuged at 10,000×g for 15 min at 4°C. The supernatant was collected, and 1 mL of supernatant was mixed with 0.25 mL of 250 g/L metaphosphoric acid solution to precipitate proteins and stabilize VFAs. After incubation on ice for 30 min, samples were centrifuged again at 10,000×g for 10 min at 4°C, and the clarified supernatant was analyzed for acetate, propionate, and butyrate using gas chromatography (GC 8890; Agilent Technologies) equipped with a DB-FFAP capillary column (30 m×0.32 mm i.d.×0.25 μm film thickness). VFA concentrations were expressed as μmol/g DM, and digesta DM was determined by oven drying at 105°C to constant weight.

### Colonic bacterial community analysis

Total genomic DNA was extracted from colonic digesta using the CTAB/SDS method. DNA concentration and purity were assessed on 1% agarose gels, and DNA was diluted to 1 ng/μL with sterile water prior to PCR amplification. The bacterial 16S rRNA geneV4 region was amplified using the primer pair 515F (5′-GTGCCAGCMGCCGCGGTAA-3′) and 806R (5′-GGACTACHVGGGTWTCTAAT-3′) with unique barcodes. PCR reactions contained 15 μL Phusion High-Fidelity PCR Master Mix (New England Biolabs), 0.2 μM of each primer, and 10 ng template DNA. Cycling conditions were 98°C for 1 min, followed by 30 cycles of 98°C for 10 s, 50°C for 30 s, and 72°C for 30 s, with a final extension at 72°C for 5 min. Amplicons were verified on 2% agarose gels, pooled in equimolar amounts, and purified using a gel extraction kit (QIAquick Gel Extraction Kit; QIAGEN).

Sequencing libraries were prepared using the NEBNext Ultra II DNA Library Prep Kit (Cat. No. E7645; New England Biolabs) according to the manufacturer’s instructions. Library quality was assessed using a Qubit 2.0 Fluorometer (Thermo Fisher Scientific) and an Agilent Bioanalyzer 2100 (Agilent Technologies). Libraries were sequenced on an Illumina NovaSeq platform to generate 250-bp paired-end reads.

Paired-end reads were assigned to samples based on unique barcodes, and barcode and primer sequences were trimmed. Reads were merged using FLASH ver. 1.2.11, quality-filtered using fastp ver. 0.20.0, and chimeras were removed against the SILVA reference database using Vsearch ver. 2.15.0. Denoising and amplicon sequence variant (ASV) inference were performed in QIIME2 (QIIME2-202006) using DADA2. ASVs with an abundance <5 were filtered out. Taxonomic assignment was conducted using the SILVA database. To minimize bias associated with sequencing depth, all samples were rarefied to an even depth corresponding to the lowest read count among samples prior to alpha- and beta-diversity analyses. Alpha diversity was assessed using the Chao1, Observed species, Shannon, and Simpson indices. Beta diversity was evaluated based on unweighted UniFrac distances, and principal coordinate analysis (PCoA) was used to visualize community differences. Group differences in community structure were assessed using analysis of similarities (ANOSIM).

Spearman’s rank correlation was used to evaluate associations between selected genera and colonic fermentation parameters (pH and VFAs). Correlation coefficients were visualized as a heatmap. Statistical significance was declared at p<0.05.

### Carcass traits and meat quality evaluation

Carcass traits were recorded following slaughter. Hot carcass weight was measured immediately after slaughter, and dressing percentage was calculated accordingly. Meat weight was determined after standardized carcass fabrication and trimming according to GB/T 27643-2011 (Technical specification for beef carcass and fresh beef fabrication), and meat yield was calculated accordingly. A representative longissimus muscle (LM) section from the left side (12th–13th rib region) was excised for subsequent measurements.

For drip loss determination, an approximately 30 g subsample was obtained from the LM, weighed, suspended in an inflated plastic bag, and stored at 4°C for 24 h; drip loss was expressed as the percentage of weight loss. The remaining LM samples were refrigerated at 4°C for 24 h prior to analysis. LM area was determined using a plastic grid, and backfat thickness was measured at the corresponding site using a caliper. Meat pH was measured using a portable pH meter (Testo 205; Testo) with a penetrating electrode. Meat color parameters (L*, a*, and b*) were measured after blooming for 30 min using a colorimeter (CR-400; Konica Minolta) standardized with a white tile. Cooking loss was determined after cooking standardized steaks to an internal temperature of 75°C, followed by cooling and reweighing. Warner–Bratzler shear force was measured on cooked cores (1.27 cm diameter) using a texture analyzer (TA.XT plus; Stable Micro Systems). For proximate composition analysis, visible fat, fascia, and external connective tissue were removed from the muscle samples before freeze-drying, and the samples were then analyzed for crude protein according to AOAC 992.15 and ether extract (intramuscular fat, IMF) according to AOAC 991.36; IMF content was expressed on a DM basis.

### Statistical analysis

All data were analyzed using SAS ver. 9.4 (SAS Institute). BW measured on d 0, 45, and 90 was analyzed as repeated measurements within animal using the PROC MIXED procedure of SAS. The model included dietary treatment, time, and their interaction as fixed effects, with animal as a random effect. Time was specified as the repeated effect within animal, and a compound symmetry covariance structure was used.

DMI was recorded repeatedly during the experimental period. Period-specific growth performance variables, including DMI, ADG, and feed efficiency for 0–45 and 46–90 d, were also analyzed using PROC MIXED. The model included dietary treatment, period, and their interaction as fixed effects, with animal as a random effect, and period was specified as the repeated effect within animal using a compound symmetry covariance structure. The statistical model was as follows:


(1) $${\text{Y}}_{\text{ijk}}=\mu +{\text{T}}_{\text{i}}+{\text{P}}_{\text{j}}+{\text{T}}_{\text{i}}\times {\text{P}}_{\text{j}}+{\text{A}}_{\text{k}}+{ɛ}_{\text{ijk}},$$

where Y_ijk_ is the observed value, μ is the overall mean, T_i_ is the fixed effect of dietary treatment, P_j_ is the fixed effect of period, T_i_×P_j_ is the interaction between dietary treatment and period, A_k_ is the random effect of animal, and ɛ_ijk_ is the residual error.

The 0–90 d values of DMI, ADG, and feed efficiency were considered whole-trial summary responses rather than additional repeated time points and were therefore analyzed separately using the GLM procedure of SAS with dietary treatment as the fixed effect.

Single-endpoint variables, including carcass traits, meat quality traits, colonic fermentation parameters, alpha-diversity indices, and the relative abundance of colonic bacterial taxa, were analyzed using the GLM procedure of SAS according to the following model:


(2) $${\text{Y}}_{\text{ij}}=\mu +{\text{T}}_{\text{i}}+{ɛ}_{\text{ij}},$$

where Y_ij_ is the dependent variable, μ is the overall mean, T_i_ is the fixed effect of dietary treatment, and ɛ_ij_ is the residual error.

Model residuals were visually inspected to assess normality and homogeneity of variance. Least squares means are presented, and when the treatment effect was significant, means were separated using Bonferroni’s multiple comparison test. For taxonomic comparisons at the phylum and genus levels, raw p values were adjusted for multiple testing using the Benjamini–Hochberg false discovery rate (FDR) procedure, and the adjusted results are presented as q values. Only taxa with q<0.05 were considered significantly different among treatments. Statistical significance was declared at p<0.05, and 0.05≤p<0.10 was considered a tendency.

## RESULTS

### Growth performance

Growth performance data are shown in Table 3. During days 0–45, DMI was greater in T2 than in CON, whereas ADG was greatest in T1 (p<0.05). During days 46–90, DMI and feed efficiency were not affected by treatment, whereas ADG tended to be higher in T1 and T2 than in CON. Across the overall 0–90 d period, both T1 and T2 had greater ADG than CON (p<0.05), and feed efficiency showed a marginal treatment effect.

### Colonic fermentation characteristics

Colonic fermentation characteristics are summarized in Table 4. Colonic pH and acetate concentration did not differ among treatments. In contrast, propionate concentration was higher in T1 and T2 than in CON (p<0.05), and butyrate concentration was higher in T2 than in CON and T1 (p<0.05).

### Colonic bacterial diversity and community structure

**Figure 1:**
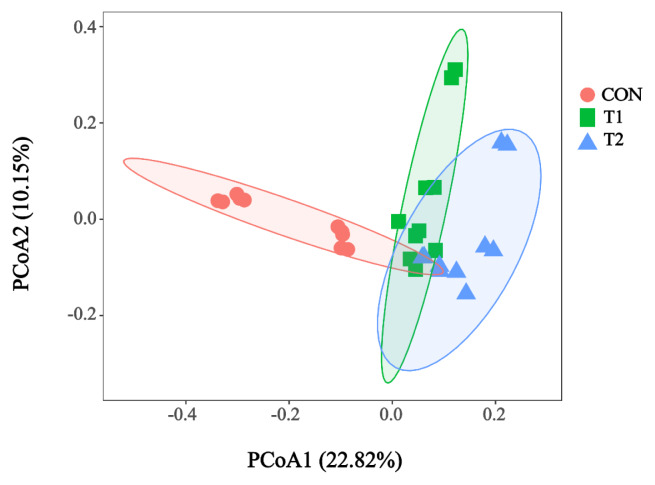
Principal coordinate analysis (PCoA) of colonic bacterial communities among treatments based on unweighted UniFrac distances. ANOSIM: R = 0.5103, p = 0.001. ANOSIM, analysis of similarities; CON, unprotected glucose (150 g/d) + coating fat (150 g/d); T1, RPG (100 g/d) + unprotected glucose (100 g/d) + coating fat (100 g/d); T2, RPG (300 g/d).

Alpha-diversity indices are presented in Table 5. No significant differences were observed among treatments in Chao1, Observed species, Shannon, or Simpson indices. However, unweighted UniFrac-based PCoA showed separation among treatments in the overall colonic bacterial community structure, and ANOSIM further confirmed a significant difference among treatments (R = 0.5103, p = 0.001) (Figure 1).

### Taxonomic composition of the colonic bacterial community

At the phylum and genus levels, Firmicutes, Prevotellaceae_UCG-003, UCG-010, and Bacteroides showed nominal differences among treatments based on raw p values. However, these differences were not retained after FDR correction (q>0.05; Tables 6, 7).

### Associations between genera and colonic fermentation parameters

**Figure 2:**
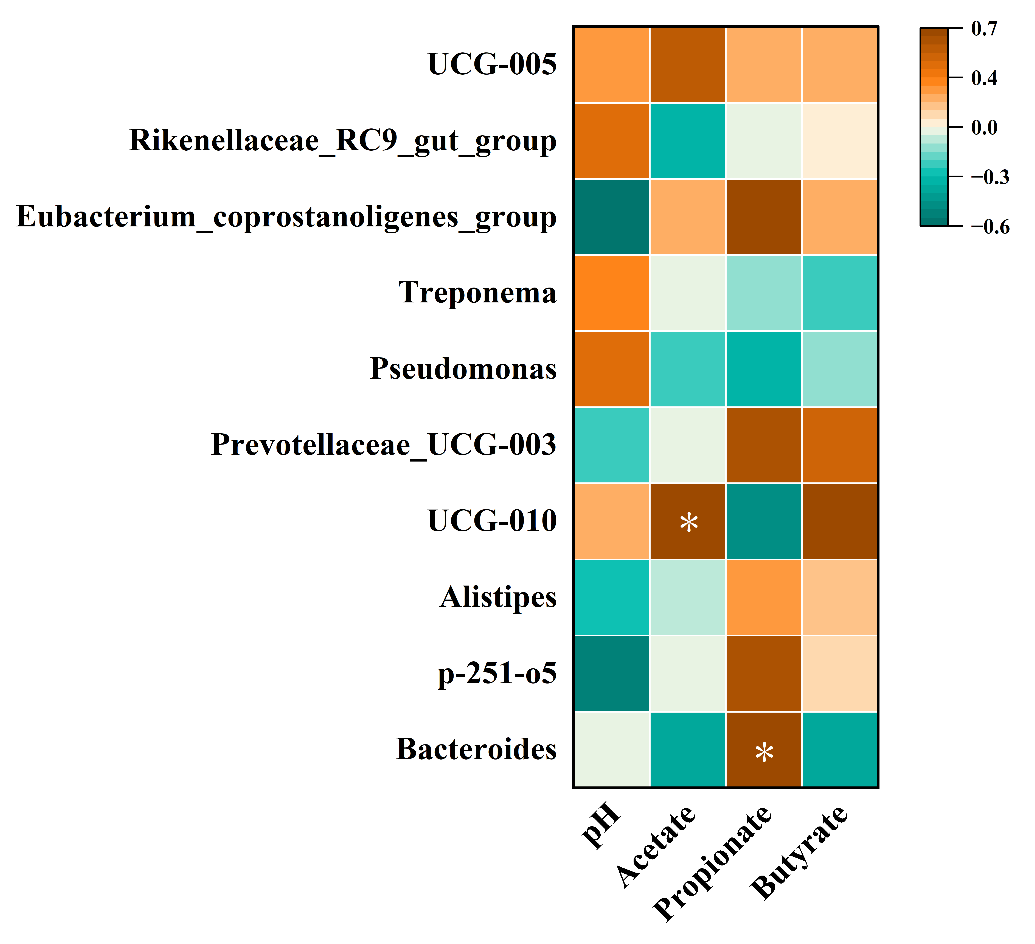
Heatmap of the correlations between colonic fermentation parameters and genus abundance. * p<0.05.

Associations between selected genera and colonic fermentation parameters are shown in Figure 2. Bacteroides was positively associated with propionate concentration, whereas UCG-010 was positively associated with acetate concentration based on raw p values.

### Carcass traits

Carcass traits are reported in Table 8. Hot carcass weight was greater in T1 and T2 than in CON (p<0.05), and meat weight was also increased in T1 and T2 compared with CON (p< 0.05). Dressing percentage and meat yield tended to be higher in T1 and T2 than in CON (p<0.10). Final live weight, LM area, and fat thickness did not differ among treatments.

### Meat quality

Meat quality traits are listed in Table 9. IMF content (Fat, %DM) differed among treatments (p<0.05), with T2 greater than CON and T1 intermediate. The other measured meat quality traits were not significantly affected by treatment, although L*, drip loss, and pH at 24 h postmortem showed tendency-level differences (p<0.10).

## DISCUSSION

In the present study, the supplementation strategies containing RPG were associated with differences in growth performance; however, the magnitude and consistency of these responses varied across feeding periods and treatments. During days 0–45, T1 showed the greatest ADG, whereas T2 mainly increased DMI relative to CON. During days 46–90, treatment effects on DMI and feed efficiency were not significant, and ADG only tended to be higher in T1 and T2 than in CON. Across the overall 0–90 d period, both T1 and T2 had greater ADG than CON, whereas the difference in feed efficiency was marginal. Therefore, the observed growth responses should be interpreted as modest and period-dependent rather than as a uniform improvement across the entire study.

Ruminants rely largely on hepatic gluconeogenesis to maintain glucose homeostasis because much of the dietary carbohydrate is fermented in the rumen. As a result, the amount of absorbable glucose reaching the small intestine is limited and may restrict anabolic metabolism under some conditions [13]. Previous studies have shown that shifting part of carbohydrate digestion from the rumen to more distal regions of the gastrointestinal tract can alter glucose metabolism and nutrient partitioning and may improve animal performance [14,15]. On this basis, RPG has been used as a nutritional approach to increase intestinal glucose supply and modify energy utilization [16]. Because the RPG product consisted of glucose and a fat-based coating, unprotected glucose and coating fat were included in CON and T1 to improve comparability among supplementation strategies. Accordingly, the treatment differences observed here were likely associated with the combined influence of supplementation level and glucose delivery pattern during finishing, rather than with glucose dose or rumen-protected delivery alone. Therefore, the present results should be interpreted as responses to the supplementation strategies evaluated.

The supplementation strategies containing RPG were associated with differences in colonic VFA profiles, as reflected by higher propionate concentrations in both RPG-supplemented groups and higher butyrate concentration in T2, whereas colonic pH and acetate remained relatively stable. Previous studies have reported that RPG supplementation may influence hindgut fermentation characteristics and microbial ecology [17,18]. The present results are generally consistent with those observations and indicate that the supplementation strategies containing RPG were associated with shifts in hindgut fermentation patterns, possibly through changes in the amount or composition of fermentable substrate reaching the colon.

Hindgut fermentation is influenced by both the quantity and characteristics of carbohydrate escaping digestion in the foregut. Therefore, changes in the site of carbohydrate utilization along the gastrointestinal tract may affect colonic VFA profiles [14,15]. In ruminants, the extent and site of starch digestion are important determinants of fermentable substrate flow to the large intestine [19]. Accordingly, dietary strategies that modify carbohydrate digestion and utilization along the gastrointestinal tract may also alter hindgut fermentation patterns [20]. In the present study, the lack of a marked change in pH suggests that the overall colonic environment remained relatively stable despite the shifts in VFA composition.

Changes in upper gastrointestinal digestion and nutrient utilization may also have influenced substrate supply to the hindgut microbiota. Previous studies have shown that glucose or glucose-yielding carbohydrate reaching the large intestine can participate in microbial fermentation and contribute to propionate formation through multiple pathways and cross-feeding interactions [21,22]. In addition, variation in propionate concentration may reflect shifts in the combined activity of several microbial pathways, even when total VFA concentration or pH changes little [23]. In the present study, differences in colonic fermentation characteristics occurred together with a shift in the overall bacterial community structure. This pattern is consistent with the possibility that the supplementation strategies were accompanied by differences in the hindgut environment, although the present data do not identify the specific microbial processes involved. Taken together, these findings indicate that the supplementation strategies containing RPG were associated with differences in hindgut fermentation characteristics and overall colonic bacterial community structure.

Unweighted UniFrac-based ordination showed separation of colonic bacterial communities among treatments, and ANOSIM further indicated a significant difference in overall community structure (R = 0.5103, p = 0.001). In contrast, alpha-diversity indices were not affected by treatment, suggesting that the response was expressed more in overall community composition than in richness or evenness. This pattern indicates that the supplementation strategies were associated with changes in the structure of the colonic bacterial community without markedly altering overall diversity.

At the taxonomic level, several phyla and genera showed nominal differences among treatments based on raw p values. However, these differences were not retained after FDR correction, indicating that the treatment-related signal was more evident at the community level than at the level of individual taxa. Thus, the microbial response observed in the present study appears to have been distributed across the overall community rather than being driven by large and robust shifts in a small number of dominant taxa. Because no taxonomic differences remained significant after FDR correction, biological interpretation at the level of specific phyla or genera is limited. Therefore, the microbiota results should be interpreted primarily as evidence of a community-level response rather than as robust shifts in specific bacterial taxa.

The correlation analysis indicated exploratory associations between selected genera and fermentation characteristics. Together with the VFA data, these findings are consistent with the possibility that treatment-related differences in the colonic environment occurred alongside changes in overall bacterial community structure. However, these associations should be interpreted cautiously as descriptive features of the community response and should not be taken as evidence of causal microbial effects on fermentation or animal performance. In this context, the microbiota results are most useful for indicating a treatment-related community-level response in the hindgut.

Overall, the microbiota findings reported here are most informative for understanding the overall colonic environment under the different supplementation strategies. It should be noted that pooling digesta from the proximal, mid, and distal colon provided an integrated representation of the colonic environment but may have obscured region-specific differences in microbial composition and fermentation characteristics.

The supplementation strategies containing RPG were associated with greater hot carcass weight and meat weight and tended to be associated with greater dressing percentage and meat yield. These results indicate that the greater overall ADG observed during the feeding period was accompanied by greater carcass output. In contrast, LM area and backfat thickness were not affected, indicating that the greater carcass weight was more likely attributable to a broadly proportional increase in tissue deposition than to a marked change in muscle accretion or subcutaneous fat deposition.

Greater availability of glucogenic nutrients beyond the rumen may support tissue accretion by increasing substrate supply for anabolic processes, which is consistent with the central role of glucose homeostasis in ruminant metabolism [13]. In addition, the site of carbohydrate digestion is an important determinant of absorbable glucose supply to the host and may therefore influence the efficiency of carcass tissue deposition [19]. On this basis, the greater carcass weight observed in RPG-supplemented bulls may be related to improved nutrient utilization during the finishing period. Overall, the present results indicate that the supplementation strategies containing RPG were associated with greater carcass output under the conditions of this study.

Most meat quality traits were not significantly affected by treatment in the present study. IMF content was higher in T2 than in CON, with T1 generally intermediate, whereas the other measured meat quality variables showed no clear treatment differences. These results suggest that the response of meat quality to the supplementation strategies was relatively limited, with the clearest effect being observed for IMF deposition.

The greater IMF observed in T2 may reflect several possible factors related to nutrient utilization and partitioning during the finishing period. A greater post-ruminal supply of glucose may have contributed to intramuscular lipid deposition, which is consistent with previous studies showing that enhanced glucose utilization can be associated with intramuscular lipid synthesis and IMF accumulation [24,25]. In addition, infusion studies have indicated that glucose may contribute proportionally more lipogenic carbon to intramuscular than to subcutaneous adipose tissue [26]. However, this interpretation remains tentative, and the higher IMF content in T2 should be viewed as a trait-specific response rather than as direct evidence of enhanced lipogenesis or a defined mechanism.

By contrast, the remaining meat quality traits were generally stable across treatments. Although L*, drip loss, and pH at 24 h postmortem showed tendency-level differences, these responses were not statistically significant and therefore should be interpreted cautiously. This overall pattern indicates that the supplementation strategies had limited influence on postmortem meat quality traits under the present conditions. Such a response is consistent with previous reports showing that ultimate pH and related traits, including meat color and water-holding capacity, are largely determined by postmortem muscle metabolism and generally remain relatively stable under normal physiological conditions [27–30]. A similar pattern has also been reported in feedlot steers supplemented with rumen-protected starch, in which marbling-related traits improved with little change in instrumental meat quality traits [31].

Overall, the present results suggest that the higher-RPG strategy (T2) was associated with greater IMF deposition, while the absence of clear changes in the remaining meat quality traits indicates that the response was mainly expressed in marbling-related characteristics.

Evidence from beef calves also indicates that targeted dietary supplementation can influence muscle development and meat quality traits, although the nutritional intervention and underlying mechanisms differ from those in the present study [32].

## CONCLUSION

The supplementation strategies containing RPG were associated with greater overall ADG, hot carcass weight, and meat weight in Simmental beef cattle. Higher colonic propionate and butyrate concentrations were observed in T1 and T2, indicating differences in hindgut fermentation among treatments. The higher-RPG strategy (T2) was also associated with greater IMF content, whereas most other meat quality traits were not clearly affected. These results should be interpreted in light of the study design, which evaluated supplementation strategies rather than the independent effects of glucose dose or delivery form.

## Figures and Tables

**Table 1:** Supplemental additive levels in the control and rumen-protected glucose (RPG) groups

	CON	T1	T2
Glucose (unprotected) (g/d)	150	100	0
RPG (g/d)^1)^	0	100	300
Coating fat (g/d)	150	100	0

1) The RPG product was composed of glucose and a fat-based coating at a 1:1 (wt/wt) ratio.

CON, unprotected glucose (150 g/d) + coating fat (150 g/d); T1, RPG (100 g/d) + unprotected glucose (100 g/d) + coating fat (100 g/d); T2, RPG (300 g/d).

**Table 2:** Ingredient composition and nutrient levels of the basal diet (DM basis)

Feed ingredients	%
Corn	45.0
Soybean meal	9.60
Distiller’s grains	23.0
Sodium bicarbonate	0.40
Limestone	0.80
Premix	0.80
Salt (NaCl)	0.40
Corn straw	20.0
Nutrient levels	
Metabolizable energy (Mcal/kg)	2.69
DM content	78.1
CP	12.0
EE	2.68
Starch	34.5
NDF	34.3
ADF	20.9
Ca	0.44
P	0.23

The premix provided the following per kg of dietary DM: vitamin A, 8,000 IU; vitamin D_3_, 1,500 IU; vitamin E, 30 IU; Zn, 50 mg; Mn, 35 mg; Cu, 10 mg; I, 0.50 mg; Se, 0.25 mg; and Co, 0.15 mg.

DM, dry matter; CP, crude protein; EE, ether extract; NDF, neutral detergent fiber; ADF, acid detergent fiber; Ca, calcium; P, phosphorus.

**Table 3:** Effects of RPG supplementation on growth performance of Simmental beef cattle

Item	CON	T1	T2	SEM	p-value
0–45 d					
Initial body weight (kg)	582.9	579.1	573.2	11.10	0.689
Final body weight (kg)	635.5	644.1	632.4	10.52	0.540
DMI (kg/d)	10.97^a^	11.03^ab^	11.43^b^	0.15	0.030
ADG (kg/d)	1.17^a^	1.44^b^	1.31^a^	0.09	0.040
Feed efficiency (ADG/DMI)	0.11	0.13	0.11	0.006	0.081
46–90 d					
Initial body weight (kg)	635.5	644.1	632.4	10.50	0.540
Final body weight (kg)	688.6	701.9	692.4	9.30	0.373
DMI (kg/d)	11.0	11.2	11.1	0.20	0.774
ADG (kg/d)	1.24	1.35	1.40	0.06	0.062
Feed efficiency (ADG/DMI)	0.11	0.12	0.13	0.004	0.150
0–90 d					
DMI (kg/d)	11.0	11.1	11.3	0.10	0.142
ADG (kg/d)	1.19^a^	1.38^b^	1.34 ^b^	0.06	0.019
Feed efficiency (ADG/DMI)	0.11	0.12	0.12	0.004	0.049

a,b Means within a row with different superscripts differ (p<0.05).

RPG, rumen-protected glucose; CON, unprotected glucose (150 g/d) + coating fat (150 g/d); T1, RPG (100 g/d) + unprotected glucose (100 g/d) + coating fat (100 g/d); T2, RPG (300 g/d); SEM, standard error of the means; DMI, dry matter intake; ADG, average daily gain.

**Table 4:** Effects of RPG supplementation on pH and VFA concentrations in colonic digesta of Simmental beef cattle

Item	CON	T1	T2	SEM	p-value
pH	6.79	6.68	6.58	0.07	0.367
Acetate (μmol/g DM)	131.57	126.57	121.60	15.33	0.219
Propionate (μmol/g DM)	31.51^a^	39.64^b^	44.26^b^	4.07	0.032
Butyrate (μmol/g DM)	12.13^a^	13.58^a^	16.23^b^	1.98	0.039

a,b Means within a row with different superscripts differ (p<0.05).

RPG, rumen-protected glucose; VFA, volatile fatty acid; CON, unprotected glucose (150 g/d) + coating fat (150 g/d); T1, RPG (100 g/d) + unprotected glucose (100 g/d) + coating fat (100 g/d); T2, RPG (300 g/d); SEM, standard error of the means; DM, dry matter.

**Table 5:** Effects of RPG supplementation on colonic bacterial alpha diversity indices in Simmental beef cattle

Item	CON	T1	T2	SEM	p-value
Chao1	1,744.0	1,717.3	1,807.7	43.7	0.185
Observed_species	1,741.7	1,696.7	1,784.3	53.5	0.330
Shannon	9.41	9.28	9.36	0.17	0.175
Simpson	0.98	0.99	0.99	0.026	0.247

RPG, rumen-protected glucose; CON, unprotected glucose (150 g/d) + coating fat (150 g/d); T1, RPG (100 g/d) + unprotected glucose (100 g/d) + coating fat (100 g/d); T2, RPG (300 g/d); SEM, standard error of the means.

**Table 6:** Relative abundance of the colonic bacterial community at the phylum level (%)

Item	CON	T1	T2	SEM	p-value	q-value
Firmicutes	54.46	58.90	52.98	2.24	0.047	0.188
Bacteroidetes	36.73	34.15	37.46	1.68	0.153	0.204
Proteobacteria	1.93	2.78	2.57	0.41	0.144	0.204
Spirochaetota	1.48	1.01	1.10	0.26	0.232	0.232

CON, unprotected glucose (150 g/d) + coating fat (150 g/d); T1, RPG (100 g/d) + unprotected glucose (100 g/d) + coating fat (100 g/d); T2, RPG (300 g/d); SEM, standard error of the means.

**Table 7:** Relative abundance of the colonic bacterial community at the genus level (%)

Item	CON	T1	T2	SEM	p-value	q-value
UCG-005	11.34	9.29	9.54	1.23	0.221	0.368
Rikenellaceae_RC9_gut_group	8.64	9.66	10.84	1.00	0.121	0.242
Eubacterium_coprostanoligenes_group	3.73	4.22	4.85	0.51	0.055	0.170
Treponema	1.32	0.92	1.02	0.16	0.076	0.369
Pseudomonas	0.30	0.23	0.31	0.05	0.362	0.369
Prevotellaceae_UCG-003	2.15	2.76	3.13	0.26	0.041	0.242
UCG-010	5.82	5.45	4.21	0.56	0.033	0.170
Alistipes	3.74	3.73	3.21	0.27	0.110	0.242
p-251-o5	0.68	0.78	0.85	0.14	0.433	0.373
Bacteroides	2.78	3.66	3.79	0.45	0.035	0.566

CON, unprotected glucose (150 g/d) + coating fat (150 g/d); T1, RPG (100 g/d) + unprotected glucose (100 g/d) + coating fat (100 g/d); T2, RPG (300 g/d); SEM, standard error of the means.

**Table 8:** Effects of RPG supplementation on carcass traits of Simmental beef cattle

Item	CON	T1	T2	SEM	p-value
Final live weight (kg)	688.6	701.9	692.4	9.30	0.373
Hot carcass weight (kg)	413.8^a^	430.3^b^	432.1^b^	6.60	0.021
Dressing percentage (%)	60.1	61.3	62.4	0.35	0.061
Meat weight (kg)	298.1^a^	315.8^b^	314.8^b^	5.34	0.015
Meat yield (%)	43.3	45.0	45.5	0.79	0.053
LM area (cm^2^)	100.1	104.0	103.7	3.91	0.347
Fat thickness (cm)	0.67	0.71	0.75	0.04	0.105

a,b Means within a row with different superscripts differ (p<0.05).

RPG, rumen-protected glucose; CON, unprotected glucose (150 g/d) + coating fat (150 g/d); T1, RPG (100 g/d) + unprotected glucose (100 g/d) + coating fat (100 g/d); T2, RPG (300 g/d); SEM, standard error of the means; LM, longissimus muscle.

**Table 9:** Effects of RPG supplementation on meat quality of Simmental beef cattle

Item	CON	T1	T2	SEM	p-value
Meat color					
L*	25.2	26.7	27.2	2.49	0.071
a*	16.9	16.6	16.6	3.15	0.995
b*	6.7	6.6	7.2	1.49	0.911
Drip loss (%)	2.40	3.28	2.83	0.30	0.069
Cooking loss (%)	36.4	35.2	34.5	1.39	0.467
Shear force (kg)	14.5	14.8	13.7	2.02	0.860
pH (24 h)	5.57	5.53	5.45	0.04	0.070
Dry matter (DM; %)	25.1	24.6	29.0	2.93	0.339
Crude protein (%DM)	77.2	78.8	78.2	0.83	0.231
Fat (%DM)	7.2^a^	7.8^ab^	8.9^b^	0.57	0.035

a,b Means within a row with different superscripts differ (p<0.05).

RPG, rumen-protected glucose; CON, unprotected glucose (150 g/d) + coating fat (150 g/d); T1, RPG (100 g/d) + unprotected glucose (100 g/d) + coating fat (100 g/d); T2, RPG (300 g/d); SEM, standard error of the means.

## Data Availability

Upon reasonable request, the datasets of this study can be available from the corresponding author.
